# Echocardiographic assessment of the relationship between cardiac function and plasma homocysteine levels in patients with heart failure and preserved ejection fraction

**DOI:** 10.3389/fcvm.2025.1525389

**Published:** 2025-03-10

**Authors:** Zi-Qi Xie, Qing Xie, Xiao-Ye Zheng, Xiao-Juan Wu, Wei-Hua Liu, Ru Li, Hong-Yan Zhu, Qi Zhou

**Affiliations:** ^1^ Medical Ultrasound Research Laboratory, The Second Affiliated Hospital of Xi'an Jiaotong University, Xi'an, Shaanxi, China; ^2^ Department of Ultrasound, Xi'an Third Hospital, The Affiliated Hospital of Northwest University, Xi'an, Shaanxi, China; ^3^ Department of Laboratory Medicine, Xi'an Third Hospital, The Affiliated Hospital of Northwest University, Xi'an, Shaanxi, China

**Keywords:** heart failure with preserved ejection fraction, homocysteine, cardiac function, myocardial remodeling, echocardiography

## Abstract

**Background:**

Heart failure with preserved ejection fraction (HFpEF) is characterized by normal ejection fraction and diastolic dysfunction. The role of plasma homocysteine (Hcy) levels in HFpEF has been understudied, though elevated levels are known to affect cardiovascular health.

**Methods:**

This retrospective observational study analyzed 80 HFpEF patients and 80 matched controls without HFpEF. Fasting plasma Hcy levels were measured using a dual-antibody sandwich enzyme-linked immunosorbent assay (ELISA). Standard echocardiographic evaluations were performed to measure interventricular septal thickness (IVST), left ventricular posterior wall thickness (LVPWT), left atrial diameter (LAD), left ventricular end-diastolic diameter (LVEDD), left ventricular ejection fraction (LVEF), and the early-to-late diastolic mitral inflow velocity ratio (E/A). Statistical analyses included independent sample t-tests, chi-square tests, Pearson's correlation, and Spearman's rank correlation.

**Results:**

HFpEF patients exhibited significantly higher plasma Hcy levels (45.17 µmol/L) compared with controls (33.85 µmol/L, *p* < 0.001). Although LVEDD and LVEF did not differ significantly between groups, HFpEF patients demonstrated increased IVST, LVPWT, LAD, and a higher E/A ratio (*p* < 0.01 for all). Plasma Hcy levels were inversely correlated with LVEF (*r* = –0.375, *p* = 0.012) and positively correlated with IVST (*r* = 0.53), LVPWT (*r* = 0.45), LAD (*r* = 0.43), and E/A ratio (*r* = 0.56; *p* < 0.01 for each). A strong positive correlation was also observed between Hcy levels and New York Heart Association (NYHA) classification (*r* = 0.824, *p* < 0.001).

**Conclusions:**

The findings indicate that elevated plasma homocysteine is associated with myocardial remodeling and impaired diastolic function in HFpEF patients. These results support the potential role of homocysteine as a biomarker for HFpEF severity and progression, warranting further investigation into its utility for risk stratification and targeted therapy.

## 1 Introduction

Heart failure with preserved ejection fraction (HFpEF) represents a complex clinical syndrome that accounts for approximately half of all heart failure cases. It is characterized by symptoms of heart failure despite a preserved ejection fraction, typically defined as an ejection fraction (EF) ≥ 50% (1–3). The pathophysiology of HFpEF is multifaceted, involving interplays between comorbidities, myocardial structure and function, and systemic inflammation. Despite its prevalence, the underlying mechanisms and effective treatment strategies for HFpEF remain inadequately understood, leading to suboptimal patient outcomes (4, 5).

A growing body of research has implicated elevated plasma homocysteine (Hcy) levels as a potential modifiable risk factor in cardiovascular diseases, including coronary artery disease and myocardial infarction. Hcy, a sulfur-containing amino acid derived from methionine metabolism, has been associated with endothelial dysfunction and increased oxidative stress, both of which play significant roles in the pathogenesis of cardiac dysfunction (6–8). Elevated Hcy levels are thought to exacerbate myocardial remodeling and fibrosis, which can impair cardiac function and contribute to the development and progression of HFpEF. Despite the known association between high Hcy levels and various forms of cardiovascular pathology, few studies have specifically investigated the relationship between plasma Hcy concentrations and cardiac function in HFpEF patients (9–11). Understanding this relationship could provide insights into the mechanisms of disease progression in HFpEF and potentially identify new therapeutic targets.

Recent studies have highlighted the potential role of plasma Hcy, a modifiable risk factor in cardiovascular diseases (12–14). Elevated Hcy levels are associated with endothelial dysfunction, oxidative stress, and inflammation, which are key contributors to cardiac remodeling and dysfunction in HFpEF (15–17). However, there remains a gap in exploring therapeutic strategies targeting Hcy, either as a direct treatment or as a biomarker for monitoring disease progression and guiding therapeutic decisions. The hypothesis of this study is that elevated plasma Hcy levels are associated with myocardial remodeling and dysfunction in HFpEF, and that Hcy may serve as a potential biomarker for disease severity and progression in HFpEF. This study aims to explore the echocardiographic characteristics of HFpEF patients and examine the correlation between plasma Hcy levels and cardiac function parameters, providing insights into the complex relationship between metabolic disturbances and cardiac function in HFpEF. These findings may inform future research and therapeutic strategies, with Hcy potentially serving as a biomarker for disease progression.

## 2 Methods

### 2.1 Study design

A retrospective observational study was conducted at our institution to assess the relationship between cardiac function and plasma Hcy levels in patients diagnosed with HFpEF. The study period spanned from January 2022 to December 2023. A total of 80 HFpEF patients were selected for detailed analysis. To establish a robust comparison, a control group of 80 patients without HFpEF was matched from the same timeframe, ensuring comparability between the two cohorts in terms of demographic and clinical characteristics. Informed consent was obtained from all subjects and/or their legal guardian(s). The research protocol, including the study design, methodology, and execution, received full approval from our hospital's ethics committee (The Second Affiliated Hospital of Xi'an Jiaotong University) and complied with international standards set forth in the Declaration of Helsinki. All procedures were carried out in strict accordance with relevant guidelines and regulations. To protect participant privacy, all personal identifiers were removed from the data, which was handled with utmost confidentiality.

### 2.2 Inclusion and exclusion criteria

Inclusion Criteria:

(1) Diagnosis of heart failure with preserved ejection fraction (HFpEF) confirmed by:
  − Clinical symptoms of heart failure (e.g., dyspnea, fatigue, or fluid retention).
  − Echocardiographic evidence of preserved ejection fraction (EF ≥ 50%).
  − Additional echocardiographic parameters consistent with HFpEF, including markers of diastolic dysfunction (e.g., E/e' ratio > 14, left atrial enlargement, or elevated tricuspid regurgitation velocity), in accordance with the latest ESC guidelines (18) or the HFA-PEFF scoring system (19).
(2) Ability to provide informed consent for participation in the study.
(3) Stable clinical condition with no changes in HF medication for at least 4 weeks prior to enrollment.
(4) Availability of complete medical records including baseline plasma Hcy levels and comprehensive echocardiographic data.

Exclusion Criteria:

(1) Patients with a history of acute myocardial infarction, percutaneous coronary intervention, or cardiac surgery within the last 6 months prior to study enrollment.
(2) Presence of significant valvular heart disease (moderate to severe valvular regurgitation or stenosis) as assessed by echocardiography.
(3) Severe renal or hepatic impairment, defined as serum creatinine >2.5 mg/dl or liver enzymes more than three times the upper limit of normal.
(4) Patients unable to undergo echocardiography due to physical limitations or poor acoustic windows.

### 2.3 Homocysteine measurement protocol using enzyme-linked immunosorbent assay (ELISA)

In our study, plasma Hcy levels are measured using a dual-antibody sandwich enzyme-linked immunosorbent assay (ELISA). The homocysteine reagent kit required for this assay was purchased from Beijing Century Ward Bio-Technology Co., Ltd. This involves collecting fasting blood samples, separating plasma, and applying it to microplates pre-coated with Hcy -specific capture antibodies. A biotinylated detection antibody and an avidin-biotin-peroxidase complex are then used to quantify Hcy levels, with optical density measured at 450 nm. Results are calibrated against a standard curve to ensure accuracy and reproducibility.

### 2.4 Echocardiographic assessment method

In our study, echocardiographic examinations are performed using a cardiac color Doppler diagnostic system. This advanced imaging technology allows for the detailed visualization and measurement of various cardiac structures and functions. In our study, echocardiographic examinations were performed using standard cardiac color Doppler techniques. These methods allow for the detailed visualization and measurement of key cardiac structures and functions. The specific parameters assessed include:

(1) Interventricular Septal Thickness (IVST): The thickness of the wall separating the left and right ventricles.
(2) Left Ventricular Posterior Wall Thickness (LVPWT): The thickness of the outer wall of the left ventricle.
(3) Left Ventricular End-Diastolic Diameter (LVEDD): The internal diameter of the left ventricle at the end of the diastole.
(4) Left Atrial Diameter (LAD): The diameter of the left atrium.
(5) Left Ventricular Ejection Fraction (LVEF): A measurement of how much blood the left ventricle pumps out with each contraction, expressed as a percentage.
(6) Early Diastolic Mitral Inflow Velocity (E) to Late Diastolic Mitral Inflow Velocity (A) Ratio (E/A Ratio): This ratio assesses diastolic function by comparing the blood flow velocities across the mitral valve during the early (E) and late (A) phases of diastole.

### 2.5 Data collection

The data for this study were retrospectively collected from the electronic medical records system of our Hospital. The dataset included detailed demographic information (age, sex), clinical comorbidities (hypertension, coronary artery disease, diabetes mellitus, atrial fibrillation, smoking status), and heart failure classification based on the New York Heart Association (NYHA) functional classification (Class II and Class III). Additionally, echocardiographic parameters such as LVEDD, LVEF, IVS thickness, LVPW thickness, LAD, and the E/A ratio were collected. Plasma Hcy levels were measured and recorded for all participants. Data collection was performed in strict compliance with institutional ethical guidelines, ensuring patient confidentiality by removing personal identifiers from all records. No clinical trial registration was required as this was a retrospective study based solely on historical medical records.

### 2.6 Statistical analysis

All statistical analyses were conducted using SPSS software (Version 27.0). Normality of the data was assessed prior to performing further statistical tests. For quantitative variables that conformed to a normal distribution, differences between groups were evaluated using independent sample t-tests, with results expressed as mean ± standard deviation. Categorical variables were presented as frequencies and percentages, and associations among these variables were analyzed using Chi-square (*χ*^2^) tests. Pearson's correlation coefficient was used to assess linear relationships between key variables, including plasma homocysteine levels and echocardiographic measurements. Normality was confirmed using the Shapiro–Wilk test, and since all variables were normally distributed, Pearson's method was deemed appropriate. All tests were two-tailed, and statistical significance was set at *p* < 0.05.

## 3 Results

### 3.1 Patient demographics and clinical characteristics in HFpEF and control groups

The study enrolled 80 patients in the heart failure with HFpEF group and 80 patients in the control group. The HFpEF group consisted of 43 males and 37 females, with ages ranging from 53 to 81 years (mean age 62.8 ± 7.8 years). Comorbidities in the HFpEF group included hypertension in 41 patients, coronary artery disease in 15 patients, diabetes mellitus in 21 patients, atrial fibrillation in 10 patients, and 25 were current smokers. In terms of severity of heart failure symptoms, according to the NYHA classification, 51 patients were classified as Class II and 29 as Class III. The control group, designed to match the HFpEF group demographically and clinically, comprised 41 males and 39 females, with an age distribution from 55 to 83 years (mean age 63.5 ± 7.5 years). This group had a slightly different distribution of comorbidities: 39 patients with hypertension, 16 with coronary artery disease, 19 with diabetes mellitus, 11 with atrial fibrillation, and 26 smokers. NYHA classification for the control group showed 53 patients at Class II and 27 at Class III (Table 1). Statistical analysis revealed no significant differences between the two groups in terms of age, sex, prevalence of comorbid conditions, or smoking habits, with all *p*-values exceeding the 0.05 threshold.

**Table 1:** Demographic and clinical characteristics of hFpEF and control groups.

Characteristic	HFpEF group (*n* = 80)	Control group (*n* = 80)
Sex		
Male	43	41
Female	37	39
Age (mean ± SD)	62.8 ± 7.8	63.5 ± 7.5
Comorbidities		
Hypertension	41	39
Coronary artery disease	15	16
Diabetes mellitus	21	19
Atrial fibrillation	10	11
Smoking	25	26
NYHA classification		
Class II	51	53
Class III	29	27

SD, standard deviation.

### 3.2 Differential plasma homocysteine levels in HFpEF compared to control group

**Figure 1:**
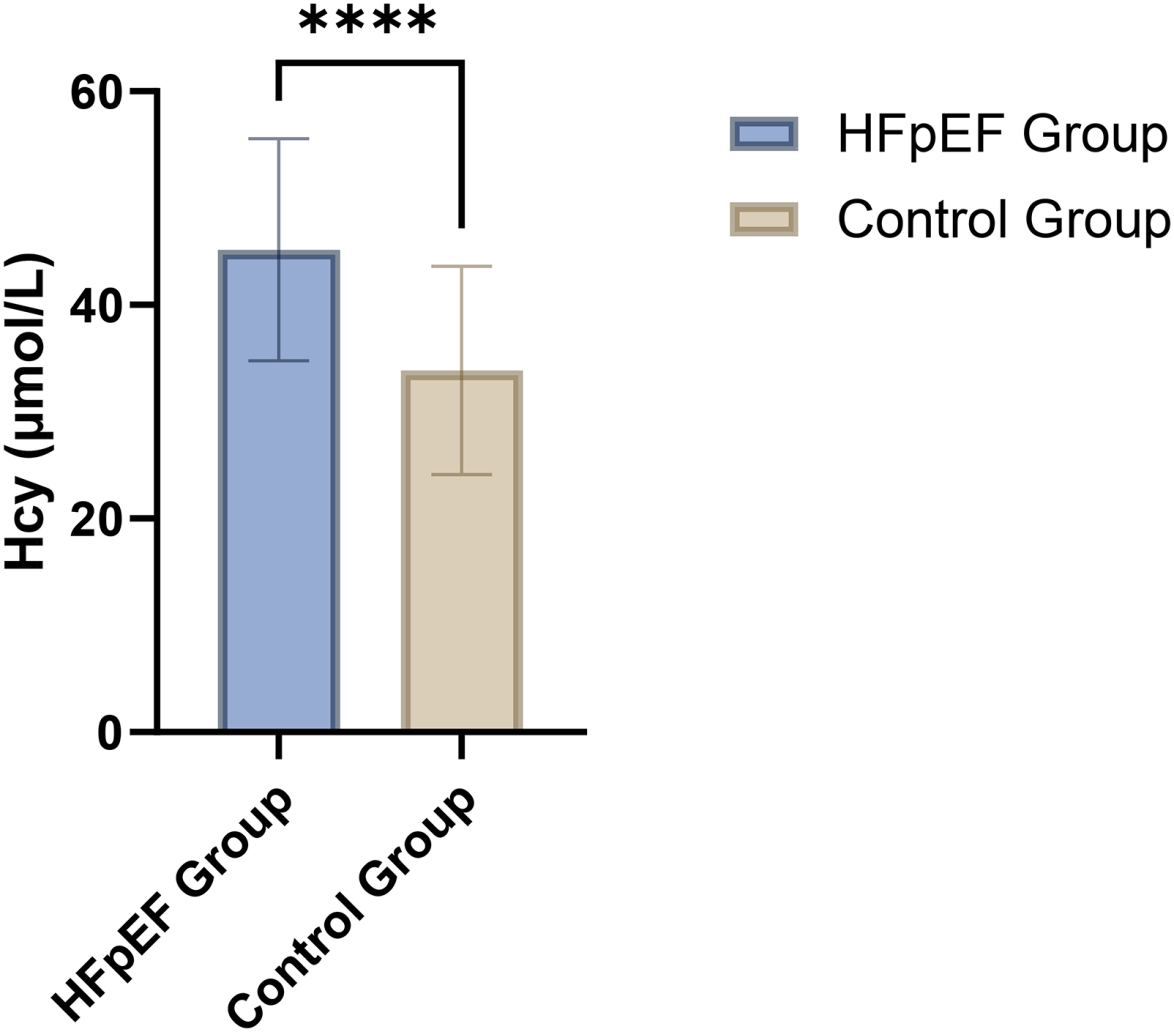
Comparison of plasma homocysteine levels between HFpEF patients (*n* = 80) and the control group (*n* = 80).

The analysis of plasma Hcy levels revealed a significant elevation in patients with heart failure with HFpEF compared to the control group. Specifically, the HFpEF group exhibited a mean Hcy concentration of 45.17 µmol/L, which was substantially higher than the 33.85 µmol/L observed in the control group. This difference was statistically significant, as indicated by a *t*-value of 7.123 and a *p*-value of less than 0.001 (Figure 1; Table 2). This marked disparity in Hcy levels underscores the potential role of this biomarker in the pathophysiology of HFpEF and suggests that elevated Hcy could be linked to the mechanisms contributing to cardiac dysfunction in this patient population.

**Table 2:** Comparison of plasma homocysteine levels between HFpEF patients and control group.

Plasma indicators	HFpEF group (*n* = 80)	Control group (*n* = 80)	*t*-value	*p*-value
Hcy (μmol/L)	45.17 ± 10.42	33.85 ± 9.75	7.123	<0.001

Hcy, homocysteine; HFpEF, heart failure with preserved ejection fraction.

### 3.3 Comparative analysis of cardiac function and myocardial remodeling in HFpEF and control groups

In this study, we evaluated cardiac function and myocardial remodeling indicators across two groups: patients with heart failure with HFpEF and a matched control group. Our analysis focused on key echocardiographic parameters including LVEDD, LVEF, thicknesses of the IVS and LVPW, LAD, and the E/A ratio which measures diastolic function. The results demonstrated no significant differences in LVEDD (45.30 ± 6.00 mm in HFpEF vs. 46.80 ± 7.00 mm in controls; *p* = 0.255) and LVEF (59.80 ± 5.40% vs. 61.00 ± 6.00%; *p* = 0.24) between the two groups, suggesting similar systolic and global diastolic dimensions and function. However, significant differences were noted in the structural parameters that are indicative of myocardial remodeling. Specifically, the HFpEF group showed increased thickness of the IVS (12.10 ± 1.00 mm vs. 11.50 ± 0.95 mm; *p* < 0.01) and LVPW (11.40 ± 1.10 mm vs. 10.50 ± 0.85 mm; *p* < 0.01), as well as a larger LAD (32.20 ± 2.20 mm vs. 29.80 ± 2.40 mm; *p* < 0.01), compared to the control group (Table 3). Additionally, the HFpEF group exhibited a higher E/A ratio (1.15 ± 0.21 vs. 0.96 ± 0.18; *p* < 0.01), indicating altered diastolic filling patterns that are often seen in myocardial stiffness and remodeling associated with HFpEF.

**Table 3:** Comparison of cardiac function and myocardial remodeling indicators between two groups of patients.

Group	LVEDD (mm)	LVEF (%)	IVST (mm)	LVPWT (mm)	LAD (mm)	E/A ratio
HF-PEF Group	45.30 ± 6.00	59.80 ± 5.40	12.10 ± 1.00	11.40 ± 1.10	32.20 ± 2.20	1.15 ± 0.21
Control group	46.80 ± 7.00	61.00 ± 6.00	11.50 ± 0.95	10.50 ± 0.85	29.80 ± 2.40	0.96 ± 0.18
*t* value	1.25	1.15	4.6	5	5.5	5.2
*P* value	0.255	0.24	<0.01	<0.01	<0.01	<0.01

LVEDD, left ventricular end-diastolic diameter; LVEF, left ventricular ejection fraction; IVST, interventricular septal thickness; LVPWT, left ventricular posterior wall thickness; LAD, left atrial diameter; E/A, ratio of early (E) to late (A) ventricular filling velocities.

### 3.4 Correlations between plasma homocysteine levels and cardiac remodeling parameters in HFpEF patients

**Figure 2:**
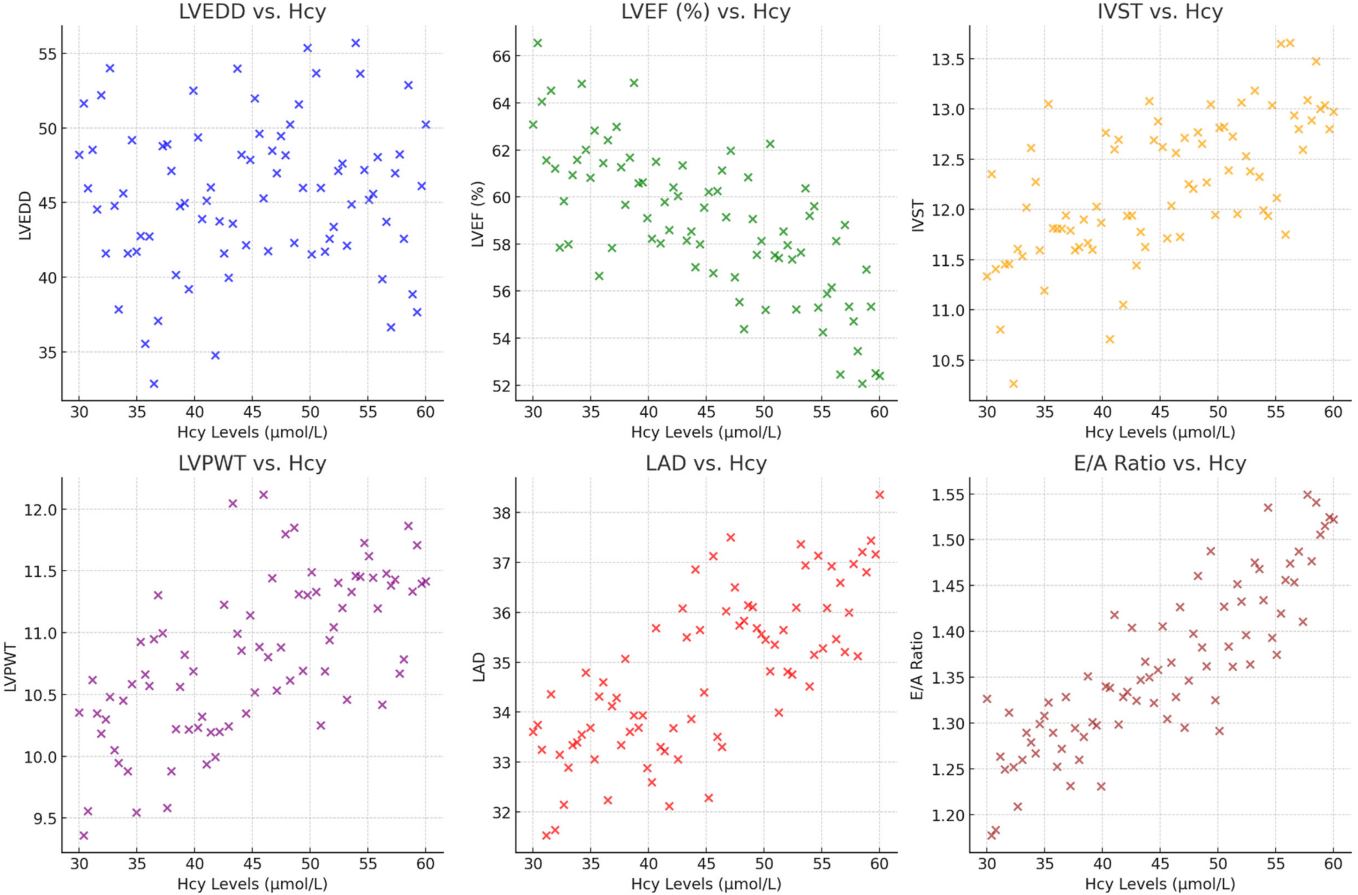
The scatter plots show the relationship between plasma Hcy levels and various cardiac indicators, including LVEDD, LVEF (%), IVST, LVPWT, LAD, and the E/A ratio.

We observed that plasma Hcy levels had a negative correlation with LVEF, indicating that higher Hcy levels were associated with worse systolic function (*r* = −0.375, *p* = 0.012). Conversely, significant positive correlations were found between Hcy levels and measures indicative of myocardial remodeling. These included IVS thickness (*r* = 0.53, *p* < 0.01), LVPW thickness (*r* = 0.45, *p* < 0.01), and LAD (*r* = 0.43, *p* < 0.01). Each of these relationships suggests that elevated Hcy levels may be linked to increased cardiac muscle mass and atrial size, both markers of pathological cardiac remodeling. Moreover, Hcy levels also showed a strong positive correlation with the E/A ratio (*r* = 0.56, *p* < 0.01), reflecting changes in diastolic function potentially due to altered myocardial compliance or filling pressures in the context of HFpEF. No significant correlation was found with LVEDD (*r* = 0.095, *p* = 0.315), indicating that Hcy levels might not directly influence overall left ventricular size in these patients (Figure 2, Table 4). These findings suggest that plasma Hcy might play a role in the pathophysiological processes of myocardial remodeling and dysfunction in HFpEF, providing potential targets for therapeutic intervention and management of this condition.

**Table 4:** Correlation between cardiac function, myocardial remodeling indicators, and plasma Hcy in HF-PEF patients.

Indicator	LVEDD	LVEF (%)	IVST	LVPWT	LAD	E/A ratio
*r* value	0.095	−0.375	0.53	0.45	0.43	0.56
*P* value	0.315	0.012	<0.01	<0.01	<0.01	<0.01

Hcy, homocysteine; LVEDD, left ventricular end-diastolic diameter; LVEF, left ventricular ejection fraction; IVST, interventricular septal thickness; LVPWT, left ventricular posterior wall thickness; LAD, left atrial diameter; E/A, Ratio of early (E) to late (A) ventricular filling velocities.

### 3.5 Correlation between plasma homocysteine levels and heart failure severity (NYHA classification)

To assess the relationship between plasma Hcy levels and heart failure severity as classified by the NYHA functional classification system. Using Spearman's rank correlation, we found a significant positive correlation between Hcy levels and NYHA class (*r* = 0.824, *p* = 6.25e-21). This indicates that as heart failure severity progresses from Class II to Class III, there is a corresponding increase in plasma Hcy levels. These results suggest that elevated Hcy levels are closely associated with the worsening severity of heart failure in HFpEF patients, potentially reflecting its role in the pathophysiological processes underlying disease progression. This finding highlights the potential utility of Hcy as a biomarker for monitoring the severity of heart failure and its progression in clinical settings.

## 4 Discussion

HFpEF is a well-established subtype of heart failure, characterized by normal or near-normal ejection fraction alongside significant diastolic dysfunction. Patients with HFpEF exhibit symptoms related to increased left ventricular stiffness and impaired relaxation, which contribute to elevated cardiac filling pressures, particularly during physical exertion (20, 21). In our study, the significant elevation of Hcy in the HFpEF group compared to the control group is notable. This disparity underscores the importance of metabolic factors in HFpEF. Elevated Hcy is known to induce endothelial dysfunction and oxidative stress, which can contribute to the stiffening of the cardiac muscle and endothelium, ultimately leading to myocardial remodeling. Such processes are likely at play in HFpEF patients, as the cardiac muscle's ability to relax and fill efficiently during diastole is impaired. The correlations observed between high Hcy levels and increased IVS thickness, LVPW thickness, and LAD further reinforce the notion of Hcy's involvement in myocardial structural changes (22, 23). These changes are consistent with the myocardial fibrosis and hypertrophy often noted in HFpEF, potentially mediated by the pro-inflammatory and pro-fibrotic effects of Hcy. Furthermore, the negative correlation between Hcy levels and LVEF suggests a possible association between elevated Hcy and subtle impairments in cardiac function. Although systolic function as measured by LVEF is typically preserved in HFpEF, the association indicates that even within normal ranges, variations in LVEF might reflect underlying influences linked to Hcy levels. The significant positive correlation of Hcy with the E/A ratio provides insight into diastolic dysfunction in HFpEF. The E/A ratio, an echocardiographic measure of diastolic filling, indicates that higher Hcy levels are associated with altered filling patterns, perhaps due to increased left atrial pressure or changes in myocardial compliance (24). Such alterations are central to HFpEF pathophysiology, where diastolic dysfunction predominates (25, 26).

Moreover, the absence of a significant correlation between Hcy levels and LVEDD suggests that while Hcy is significantly associated with myocardial remodeling and function, it does not necessarily correlate with changes in the overall size of the left ventricle. This observation may indicate that the effects of Hcy are more closely related to the quality of myocardial tissue rather than its size, focusing on stiffness and fibrotic changes over mere hypertrophic growth. The significant positive correlation between Hcy levels and NYHA classification (*r* = 0.824, *p* < 0.001) further supports the role of Hcy as a marker of heart failure severity in HFpEF. As heart failure progresses from Class II to Class III, the increase in Hcy levels highlights its association with the worsening clinical status in HFpEF patients. These findings collectively indicate a multifaceted association between Hcy and alterations in cardiac structure and function in HFpEF (27, 28). Elevated Hcy could serve as a biomarker for disease severity in HFpEF, offering a target for potential therapeutic intervention. For instance, vitamin B supplements, known to reduce Hcy levels, might prove beneficial in mitigating myocardial remodeling and improving outcomes in HFpEF patients. The utility of Hcy as a biomarker for monitoring the progression of heart failure is further emphasized, with potential therapeutic strategies such as vitamin B supplementation, which may help mitigate the progression of myocardial remodeling and improve outcomes in HFpEF patients.

The selection of the 80 HFpEF patients was based on strict inclusion and exclusion criteria, ensuring internal validity and a homogeneous study population. Although the sample size might appear limited, the study was sufficiently powered to detect significant differences between the HFpEF and control groups. The highly significant *p*-values (*p* < 0.01) for homocysteine levels and myocardial remodeling parameters highlight the robustness of the findings. Moreover, the effect sizes observed further validate the associations between elevated homocysteine levels and cardiac remodeling in HFpEF patients. The control group was carefully matched to the HFpEF group based on demographic and clinical characteristics, including age, sex, and relevant cardiovascular comorbidities such as hypertension, coronary artery disease, diabetes mellitus, and atrial fibrillation. While these control patients did not have an HFpEF diagnosis, some exhibited mild cardiovascular symptoms justifying their NYHA classification (primarily Classes II and III). This matching process allowed for a more accurate comparison by controlling for cardiovascular risk factors, thereby clarifying the association between HFpEF, homocysteine levels, and cardiac remodeling.

Elevated Hcy levels are implicated in the pathogenesis of myocardial hypertrophy and atrial dilation through mechanisms that involve oxidative stress and endothelial dysfunction. At the molecular level, Hcy impairs endothelial function primarily by reducing the bioavailability of nitric oxide (NO), a critical mediator of vascular relaxation (29). Hcy increases the production of reactive oxygen species, which in turn oxidizes NO, diminishing its vasodilatory capacity. This reduction in NO leads to endothelial dysfunction, characterized by impaired vasodilation, increased vascular stiffness, and a pro-inflammatory state (30). Moreover, Hcy enhances the expression of adhesion molecules and pro-inflammatory cytokines, promoting leukocyte adhesion and vascular inflammation, which further exacerbates endothelial injury (31). This endothelial dysfunction is a central feature in HFpEF and may act synergistically with other HFpEF risk factors such as hypertension, diabetes mellitus, and obesity. The coexistence of these risk factors with elevated Hcy can amplify vascular inflammation and oxidative stress, thereby accelerating myocardial remodeling. Additionally, Hcy has been associated with upregulation of matrix metalloproteinases, which contribute to extracellular matrix remodeling, leading to increased myocardial wall thickness and atrial dilation (32).

This study has several limitations. First, its retrospective design may introduce selection bias and limits the ability to establish causality between elevated homocysteine levels and cardiac dysfunction in HFpEF. The single center setting further restricts the generalizability of the findings to broader populations. Additionally, reliance on available electronic medical records constrained the analysis, as factors such as stroke, cancer, and neurodegenerative diseases that might influence homocysteine levels were not included. Furthermore, the study employed conventional echocardiographic methods, which may not capture subtle aspects of myocardial dysfunction. Advanced imaging techniques, such as speckle-tracking echocardiography (e.g., strain and strain rate analysis), could provide deeper insights into myocardial mechanics and should be considered in future research.

Future research should include prospective, multicenter trials and molecular studies to explore the causal relationship between homocysteine levels and cardiac remodeling, focusing on interventions that modify Hcy levels and their impact on myocardial function. Multivariable regression models are essential to control for confounding factors and more precisely evaluate homocysteine's effects. Future research should incorporate advanced imaging techniques, biomarkers, and control groups to provide deeper insights into myocardial mechanics and enhance therapeutic strategies for HFpEF and other cardiovascular conditions. Detailed phenotyping in prospective studies is needed to explore variations in myocardial remodeling across HFpEF subgroups. Additionally, renal function should be considered as a critical factor in understanding how metabolic disturbances, including renal dysfunction, affect cardiac pathology in HFpEF.

## 5 Conclusions

Our study conclusively demonstrates that patients with HFpEF exhibit significantly elevated plasma Hcy levels compared to non-HFpEF individuals. Importantly, these elevated levels are closely associated with cardiac function and myocardial remodeling in HFpEF patients. These insights underscore the critical role of Hcy in the pathophysiology of HFpEF and its potential as a target for therapeutic intervention.

## Data Availability

The raw data supporting the conclusions of this article will be made available by the authors, without undue reservation.
